# Exploring the role of halogen bonding in iodonium ylides: insights into unexpected reactivity and reaction control

**DOI:** 10.3762/bjoc.19.86

**Published:** 2023-08-07

**Authors:** Carlee A Montgomery, Graham K Murphy

**Affiliations:** 1 Department of Chemistry, University of Waterloo, 200 University Ave W., Waterloo, Ontario, N2L3G1, Canada

**Keywords:** electron donor–acceptor complex, halogen bonding, σ-holes, iodonium ylides, *ortho*-effect

## Abstract

Halogen bonding is commonly found with iodine-containing molecules, and it arises when Lewis bases interact with iodine’s σ-holes. Halogen bonding and σ-holes have been encountered in numerous monovalent and hypervalent iodine-containing compounds, and in 2022 σ-holes were computationally confirmed and quantified in the iodonium ylide subset of hypervalent iodine compounds. In light of this new discovery, this article provides an overview of the reactions of iodonium ylides in which halogen bonding has been invoked. Herein, we summarize key discoveries and mechanistic proposals from the early iodonium ylide literature that invoked halogen bonding-type mechanisms, as well as recent reports of reactions between iodonium ylides and Lewis basic nucleophiles in which halogen bonding has been specifically invoked. The reactions discussed herein are organized to enable the reader to build an understanding of how halogen bonding might impact yield and chemoselectivity outcomes in reactions of iodonium ylides. Areas of focus include nucleophile σ-hole selectivity, and how ylide structural modifications and intramolecular halogen bonding (e.g., the *ortho*-effect) can improve ylide stability or solubility, and alter reaction outcomes.

## Introduction

Iodonium ylides are a subset of hypervalent iodine (HVI) reagents that were first reported in 1957 by Neiland [1]. These have since been investigated under a variety of thermal, photochemical, radical and transition metal-catalyzed conditions [2], and they have been successfully employed in numerous reactions such as metallocarbene chemistry [3–8], cycloadditions [9–14], and radiofluorinations [15–18]. As with most HVI reagents, reactions of iodonium ylides are often described using terminologies (e.g., ylide transfer, coupling or reductive elimination) more commonly associated with transition metal-mediated chemistry; however, halogen- or σ-hole bonding has recently emerged as a credible explanation for the diverse reactivity that iodonium ylides undergo. σ-Hole bonding theory offers a means to explain the occurrence of transition metal-free reactions in processes that are typically metal-mediated, as well as the reactions observed between iodonium ylide-Lewis base pairs, including single electron transfers and proton transfers. As iodonium ylides exhibit two σ-holes, they offer two potential sites for halogen bonding to occur, potentially resulting in two different ligand coupling outcomes. While most of their reactions expel an iodoarene to produce functionalized β-dicarbonyl motifs, ligand coupling with the arene motif is also possible, such as in the (radio)fluorination of iodonium ylides. Finally, intramolecular σ-hole bonding offers the potential to alter the physical properties (e.g., stability, solubility, UV–vis absorption) of an ylide, as well as bias a Lewis base’s σ-hole selectivity through σ-hole blocking, which represent emerging avenues for tuning an ylide’s reactivity and improving its reaction outcomes.

## Review

### 1 Halogen bonding

#### 1.1 σ-Holes in halogen bonding

The history of halogen bonding dates back to the 19th century [19–20], and yet our knowledge and understanding of this non-covalent force is continuously growing, encompassing more and more classes of organic molecules. There was no generally accepted name until it was coined “halogen bonding” by Dumas et al. in 1978 [21–22], and only in 2013 did it receive an official IUPAC definition [23]. Iodine is the largest and most polarizable of the halogens, and as such it predominantly engages in halogen bonding (I > Br > Cl >> F) [24]. Halogen bonding was first observed by Guthrie in the 1860s as an attraction between ammonia and molecular iodine [19]. The 20th century saw halogen bonding extended to organohalides such as diiodoacetylene [25–26], and also saw the first X-ray crystallographic evidence of a ‘halogen molecular bridge’ between molecular bromine and 1,4-dioxane [27–28]. In 1953, the first instance of halogen bonding in a hypervalent iodine-containing molecule was reported [29], and this set the stage for decades of subsequent investigation.

**Figure 1:**
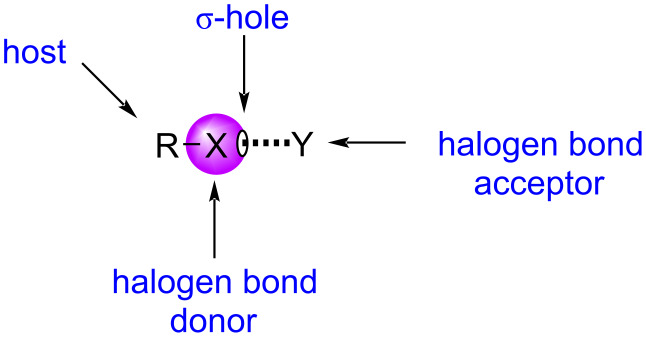
Generic representation of halogen bonding.

To fully understand halogen bonding, a thorough understanding of the properties of σ-hole bonding is necessary. A σ-hole bond is the non-covalent inter- or intramolecular interaction between the σ-hole of a group IV–VII atom with the electron-rich site of Lewis bases such as anions, hydrides, or even π electrons [24,30]. Halogen bonding is a subset of this bonding classification, represented by the generic bonding pattern shown in Figure 1, where R is the host atom or functional group to which the halogen is covalently bound, where X is the halogen atom possessing the σ-hole (halogen bond donor), and where Y is the Lewis base (halogen bond acceptor) [31].

σ-Holes arise from anisotropic covalent bonds between the halogen and its host, where polarization of the halogen’s electronic charge is directed towards the covalent bond [24]. This results in a phenomenon called polar flattening, resulting in a non-spherical distribution of electron density on the halogen’s surface, with a positive electrostatic potential located along the extension of the covalent bond, and with a lateral belt of negative electrostatic potential [31–35]. The resulting region of diminished electron density is dominated by the electrostatic contributions from the nucleus over those from its electrons and results in a σ-hole [24,36–39]. This anisotropic electron distribution manifests itself in many ways, such as the L-shaped packing observed with diatomic halogens resulting from the secondary bonding between the positive end of one halogen and the negative end of another [40].

σ-Holes can be assessed both qualitatively and quantitatively, enabling predictions of their properties and comparisons of their non-covalent halogen bonding interactions [41]. A molecular electrostatic potential (MEP) map, taken as the 0.001 au density envelope, is typically used for qualitative σ-hole depictions [42–43]. Conversely, the electrostatic potentials at specific points on the atom’s surface can be quantitatively determined through either computation or diffraction techniques [44–45]. Computation of the surface’s electrostatic potential extrema (*V*_S,max_ and *V*_S,min_) are based on Equation 1 [31], which considers the contributions of both the nucleus and the electrons at a point in space (*r*), where *Z*_A_ is the charge on nucleus A, located at *R*_A_, and where ρ(*r*) is the molecule’s electron density. A positive electrostatic potential (*V*_S,max_) value signifies a dominating nuclear contribution and a positive σ-hole.


[1] $$V(r)=\sum_{\text{A}}\frac{Z_{\text{A}}}{\left|R_{\text{A}}-r\right|}-\int \frac{\rho (r')dr'}{\left|r'-r\right|}$$

The electrostatic strength of a σ-hole typically increases with polarizability within the same group (I > Br > Cl >> F) [41,46–47]; however, the electron attracting abilities of the host [31,46], the electronegativity of the halogen atom [41] and, the degree of sp*^n^*-hybridization in the covalent σ_R–X_ bond can also influence σ-hole strength [32,48]. Should the halogen possess two covalent bonds, two σ-holes would result with the stronger situated opposite the more electron-attracting group [24]. σ-Holes are also highly directional, with near linear halogen bond-acceptor approach angles that typically fall between 160–180° [39,49–50], and with halogen bond lengths that are typically less than or equal to the sum of the atomic Van der Waals radii of the engaged atoms [33]. The strength of a subsequent halogen bond is influenced by the magnitudes of the positive electrostatic potential (*V*_S,max_) of the donor and the negative electrostatic potential (*V*_S,min_) of the acceptor [46,48,51–52], but also by other factors [53–57] including hydrogen bonding [31,48], solvent polarity [47,58], the Lewis basicity of the acceptor [46,50] and by sterics [46]. As with any interatomic interaction, the energy of a halogen bond is influenced by multiple factors including polarization, electrostatic attraction, charge transfer, dispersion and molecular orbital interactions [59–63]. The charge transfer nature (donation into the σ* orbital of the R–X bond) of the halogen bond leads to elongation and weakening in the host/donor R–X bond, and an accompanied decrease in the HOMO–LUMO gap which may be experimentally observed as a red-shift in the vibrational frequency [46–47,64–65].

#### 1.2 Halogen bonding in monovalent iodine

**Figure 2:**
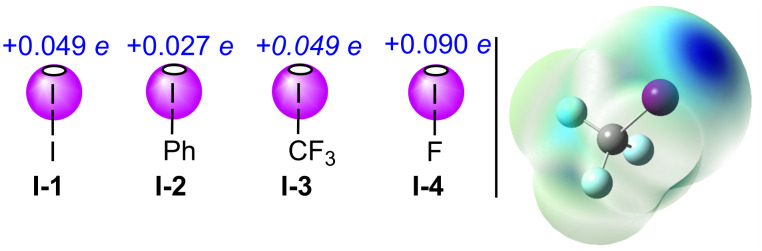
Quantitative evaluation of σ-holes in monovalent iodine-containing compounds; and, qualitative molecular electrostatic potential (MEP) of **I-3** from −0.052 to 0.052 *e* showing a single σ-hole (dark blue) [86].

The σ-holes expressed by both molecular iodine and other organoiodine compounds have been evaluated extensively through both qualitative and quantitative means. Methods of evaluation have included X-ray crystallography and spectroscopy (e.g., microwave, IR, Raman, NMR, NQR), as well as through computational determination of their electrostatic V_S,max_ potentials (Figure 2), their halogen bond lengths and their interaction energies [66–71]. Comparing the *V*_S,max_ of select iodine-containing molecules shows that the electrostatic potential for molecular iodine (**I-1**) was 0.049 *e*, significantly greater than that of iodobenzene (**I-2**, 0.027 *e*) [70,72], though identical to that of trifluoroiodomethane (**I-3**, 0.049 *e*) [70–71]. As expected, given the ability of the host atom to directly influence the strength of the σ-hole, a significantly increased V_S,max_ value of 0.090 *e* was observed for iodine monofluoride (**I-4**) [72]. Since halogen- and hydrogen bonding exhibit numerous similar attributes, halogen bonding has also proven to be a viable alternative in methodologies that rely on hydrogen bond initiation. For example, both halogen- and hydrogen bonding can be used in supramolecular chemistry as the binding mechanism in photoresponsive receptors [73–82]. Organocatalysis is also a common point of intersection for halogen- and hydrogen bonding, and this has been thoroughly explored using monovalent iodine catalysts [83–85].

#### 1.3 Halogen bonding in hypervalent iodine complexes

**Figure 3:**
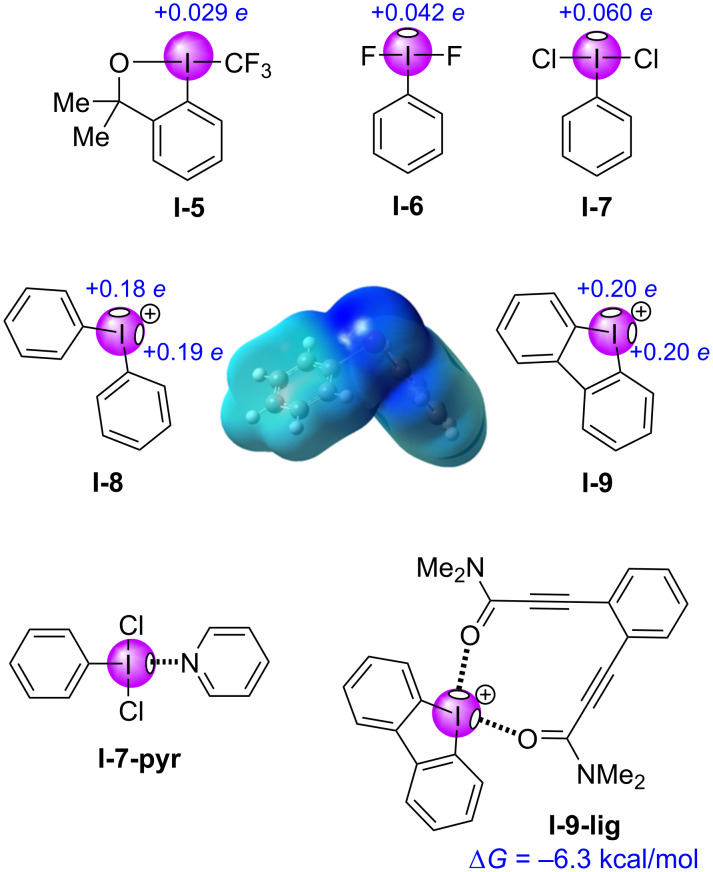
Quantitative evaluation of σ-holes in hypervalent iodine-containing molecules; and, qualitative MEP map of **I-8** from −0.199 to 0.199 *e* showing two σ-holes (dark blue) [86].

Similar to monovalent iodine compounds, a diverse selection of hypervalent iodine compounds have also been assessed in silico to determine the strengths of their σ-holes (Figure 3). Togni’s CF_3_-benziodoxole reagent (**I-5**, 0.029 *e*) possessed the weakest σ-hole from among those analyzed, consistent with monovalent iodobenzene (**I-2**) [72]. Difluoroiodobenzene (**I-6**, 0.042 *e*) and dichloroiodobenzene (**I-7**, 0.060 *e*) showed similar potentials with **I-2** and **I-3**, with the weaker σ-hole found on the more covalent difluoroiodoarene species **I-6** [72,87]. The symmetric diaryliodonium salts **I-8** and **I-9** each presented two σ-holes, with the acyclic variant’s potentials being slightly different (0.18 *e* and 0.19 *e*) due to its non-planarity, whereas the cyclic, planar variant’s potentials were each 0.20 *e* [88–89]. The strength of the σ-holes for **I-8** and **I-9** were far greater than those of other HVI reagents, which was unsurprising given that its iodine atom was positively charged. Halogen-bonded adducts of these have also been observed, which further illustrated the existence and properties of σ-holes in such HVI compounds. For example, **I-7-pyr** was characterized by X-ray crystallography and by computational methods, where molecular orbital (MO) analysis showed that pyridine was in fact interacting with the LUMO+1 MO of **I-7**, corresponding to the σ* orbital oriented along the I–C bond axis [87]. Lüthi et al. quantified the symmetry-adapted perturbation theory (SAPT) interaction energies of halogen bonded acetonitrile complexes of HVI molecules [72], and Huber et al. used ^1^H NMR titrations and isothermal titration calorimetry (ITC) to experimentally determine interactions energies for **I-9** complexes (e.g., **I-9-lig**, −6.3 kcal/mol), they also discovered that simultaneous binding to both σ-holes was possible [90]. Given these observations, it is unsurprising that HVI compounds have also played an active role in halogen bond-based organocatalysis [91–97].

### 2 Halogen bonding in iodonium ylides

#### 2.1 Quantification of σ-holes in iodonium ylides

**Figure 4:**
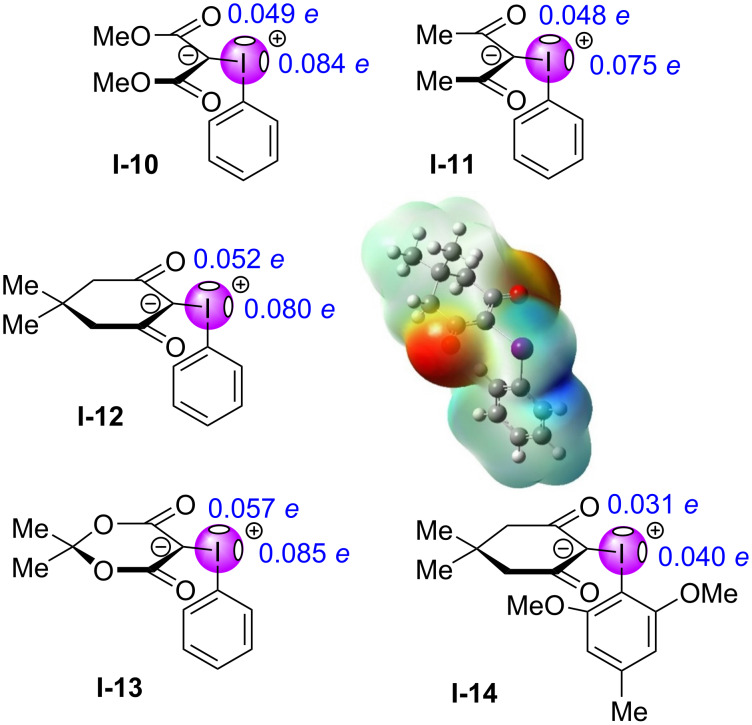
Quantitative evaluation of σ-holes in iodonium ylides; and, qualitative MEP map of **I-12** from −0.083 to 0.083 *e*, showing strong (dark blue) and weaker (light blue) σ-holes [86].

Until recently, there were no quantitative evaluations of σ-holes in iodonium ylides [98–99], even though halogen bonding had been invoked for years when describing reactions of such ylides. In 2022, Murphy et al. used computational analysis to assess iodonium ylides for their σ-holes, and found that two of these existed, with one situated opposite the arene and the other opposite the β-dicarbonyl motif (Figure 4) [100]. In the dimethyl malonate-derived ylide **I-10**, the σ-hole opposite the β-dicarbonyl was stronger with an electrostatic potential of 0.084 *e*, compared to the 0.049 *e* found for that opposite the arene. The same pattern was observed for the acetylacetone-derived ylide **I-11**, which possessed potentials of 0.075 *e* and 0.048 *e*. The cyclic dimedone-derived ylide **I-12** showed similar potentials of 0.080 *e* and 0.052 *e*, as did the Meldrum’s acid-derived ylide **I-13** (0.085 *e* and 0.057 *e*). Whether the ylide was cyclic or acyclic, the σ-hole opposite the β-dicarbonyl motif was consistently greater in strength due to its higher electron affinity. While this trend was also true with the poly-substituted ylide **I-14**, the electron-rich arene significantly offset the electropositivity on the iodine, resulting in two σ-holes of diminished strength (0.040 *e*, 0.031 *e*).

**Scheme 1:**
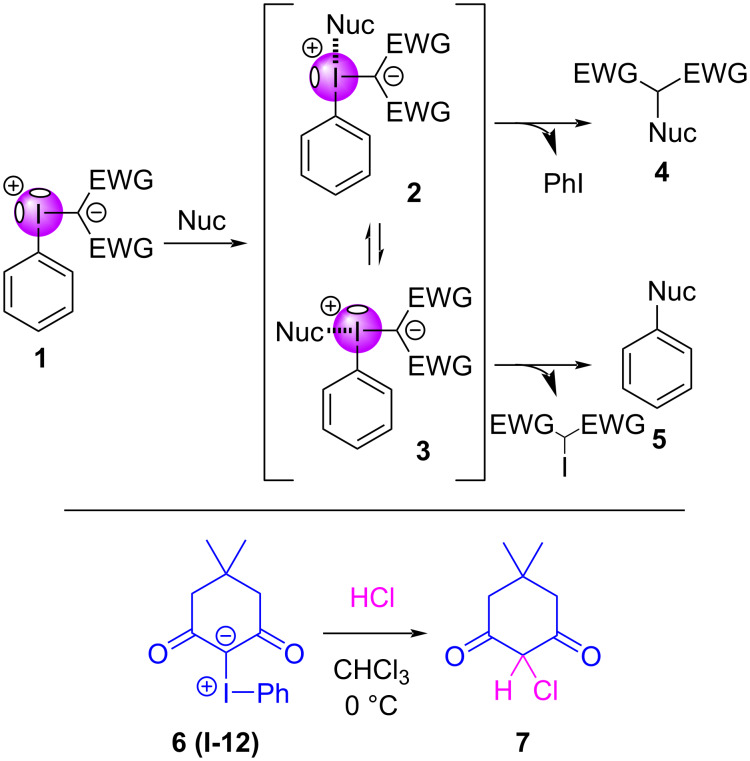
Outline of possible reaction pathways between iodonium ylides and Lewis basic nucleophiles (top); and, reaction between **6** and HCl to give **7**, as an example of β-dicarbonyl functionalization.

Despite the historically limited appreciation and understanding of σ-holes in iodonium ylides, many examples exist where inter- or intramolecular halogen bonding has been invoked to provide a meaningful explanation of unexpected reactivity, of reagent stability, or as a reaction control element. Examples of these include spontaneous cycloaddition reactions that occur without transition metal catalysts, the unexpected initiation of single electron transfer (SET) processes or photochemical transformations, and even proton transfers that appear to defy p*K*_a_ limitations. The reaction pathways followed by iodonium ylides and Lewis basic reaction partners, from the initial adducts they form to the different products they produce, has long been a cause for discussion. This concept was elegantly illustrated by Kobayashi and Takemoto (Scheme 1) [101], who suggested that an ylide **1** may halogen bond with a nucleophile using either of its σ-holes to generate intermediates **2** or **3**, where the *cis*-oriented ligands may reductively eliminate to generate either a functionalized β-dicarbonyl (**4**, most common) or a substituted arene (**5**, rare). Interestingly, when the first iodonium ylide was synthesized by Neiland and co-workers in 1957, they also evaluated its decomposition [1,102]. They discovered that an iodonium ylide (e.g., **6**/**I-12**) reacts with hydrochloric acid to produce a chlorinated β-dicarbonyl **7**, opposite their expectation that the C–I bond with the lower electron density would be cleaved.

While both of these reaction pathways are viable, the iodonium ylide literature to-date has shown that the vast majority of reactions follow the reductive elimination pathway to produce **4**. However, a clear exception to this is in reactions with nucleophilic fluoride or [^18^F]fluoride, which reductively eliminate to exclusively generate fluoroarenes (e.g., **5**). The rationale for these preferential outcomes is not understood, and may derive from rapidly equilibrating species (e.g., **2** and **3**) reacting under Curtin–Hammett control, from hard/soft principles dictating the Lewis base’s selectivity with either the stronger (e.g., **3**) or weaker (e.g., **2**) σ-hole, or possibly from other contributing factors.

#### 2.2 Halogen bond-initiated reactions: early discoveries

**Scheme 2:**
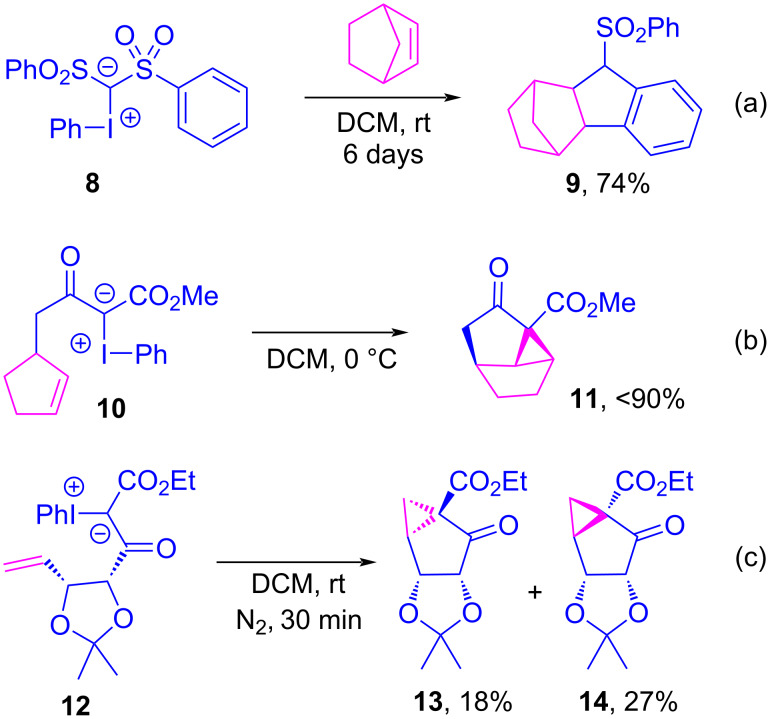
Metal-free cyclopropanations of iodonium ylides, either as intermolecular (a) or intramolecular processes (b, c).

Iodonium ylides have long been employed as metallocarbene precursors, and Müller et al. contributed significant evidence that their reactions with olefins took place within the coordination sphere of the metal [103–106]. However, numerous inconsistencies have been observed when comparing the outcomes of diazo- and iodonium ylide-based metallocarbene reactions, especially during metal-free control experiments, which led researchers to propose alternative, carbene-free reaction pathways for iodonium ylides. This was first reported by Hadjiarapoglou, Varvoglis and co-workers [107–112] and Moriarty et al. [113–115], who observed metal-free cycloadditions between iodonium ylides and olefins in the absence of a transition metal catalyst. These reactions proceeded under mild conditions without catalyst, where the byproducts typically associated with free carbene formation (e.g., dimerization or rearrangement) were not observed (Scheme 2). In 1988, Hadjiarapoglou was investigating transition metal- and photocatalyzed intermolecular cyclopropanations between ylide **8** and norbornene, and found indane **9** was produced in 74% yield after six days at room temperature in the absence of catalyst (Scheme 2a) [107]. At that time, the authors did not articulate any theory as to how the reaction might initiate under such mild conditions, though they recognized that the decomposition of **8** into a free carbene would not have been feasible. In 1989, Moriarty was investigating the intramolecular cyclopropanation of **10** under copper-catalysis, presuming that the reaction would proceed through a metallocarbene intermediate [113]. However, a control experiment showed the reaction to also be viable without catalyst, from which the expected product of a free carbene-derived Wolff-type rearrangement was not observed (Scheme 2b). Likewise, Gallos et al. were investigating the intramolecular cyclopropanation of ylide **12**, and found that the metal-free reaction proceeded with identical yield and diastereoselectivity as did their rhodium- and copper-catalyzed reactions (Scheme 2c) [116]. These results were in stark contrast to those observed for the ylide’s diazo counterparts, which did not react without a catalyst, and which gave the opposite diastereoselectivity with copper and rhodium.

**Figure 5:**
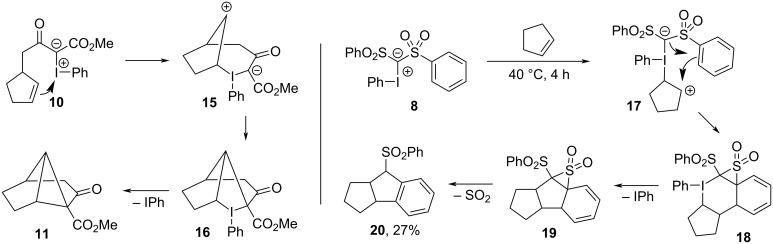
Zwitterionic mechanism for intramolecular cyclopropanation of iodonium ylides (left); and, stepwise intermolecular cycloadditions with olefins (right).

Inspired by ionic pathways previously proposed for hypervalent iodine compounds [9,117–119], Moriarty proposed a carbene-free mechanism for the formation of **11** [113]. In this, the nucleophilic olefin first attacked the electrophilic iodine center to form zwitterion **15**, which closed to produce iodocycle **16** and then underwent reductive elimination of iodobenzene to give **11** (Figure 5, left). This was also the pathway subsequently suggested by Gallos et al. [116] for the synthesis of **13**/**14** from **12**. In 2003, Hadjiarapoglou further investigated the intermolecular reaction between **8** and cyclopentene under thermal, metal-free conditions, and offered a related mechanistic proposal (Figure 5, right) for the formation of **20** [110]. Given that their reaction proceeded almost equally well under metal-free conditions as under Rh_2_(OAc)_4_ catalysis (3.5 h, 40 °C, 31% **20**), and that free carbene formation was unlikely at this temperature, they believed that the reaction was likely initiated by either single electron transfer between the reagents (not shown), or by electrophilic addition of the olefin onto the ylide, forming intermediate adduct **17**. This was followed by formation of iodocycle **18**, from which reductive elimination of iodobenzene gave **19**, and extrusion of SO_2_ gave **20**.

**Scheme 3:**
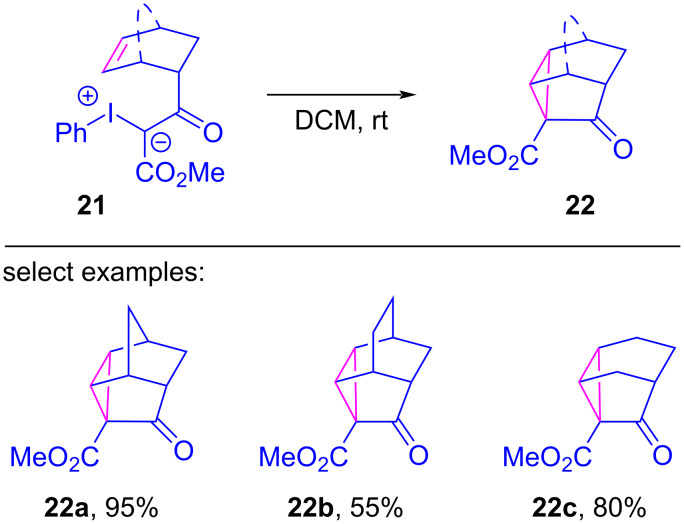
Metal-free intramolecular cyclopropanation of iodonium ylides.

**Figure 6:**
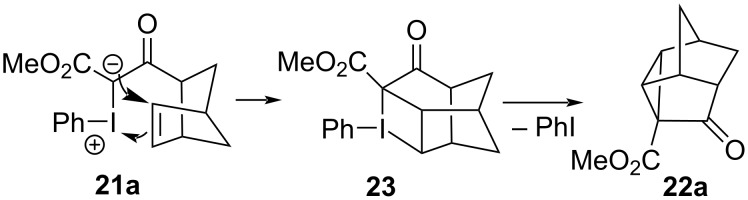
Concerted cycloaddition pathway for the metal-free, intramolecular cyclopropanation of iodonium ylides.

In 2010, Moriarty revisited their earlier transformation and reported that the metal-free intramolecular cyclopropanation of **10** to **11** could be achieved in an improved 95% yield by performing the reaction at room temperature rather than 0 °C [11]. The reaction was also viable with a selection of other mono- and bicyclic olefin motifs, undergoing intramolecular cyclopropanations in moderate to high yields (Scheme 3). They also conducted a mechanistic investigation to better understand this metal-free pathway, again on the premise that a free carbene intermediate was not viable under such mild conditions. The initially proposed ionic pathway (Figure 5, left) was abandoned as solvent effects had little influence on the reaction rate, and since no Wagner–Meerwein rearrangement products were detected with bicyclic olefin precursors. Radical-based pathways were also discredited because ESR spectra of reaction mixtures showed no evidence of unpaired electrons. Instead, a concerted cycloaddition pathway was proposed for the formation of iodocyclobutane **23** (Figure 6), which would again be followed by reductive elimination of iodobenzene to give the cyclopropane **22a**.

**Scheme 4:**
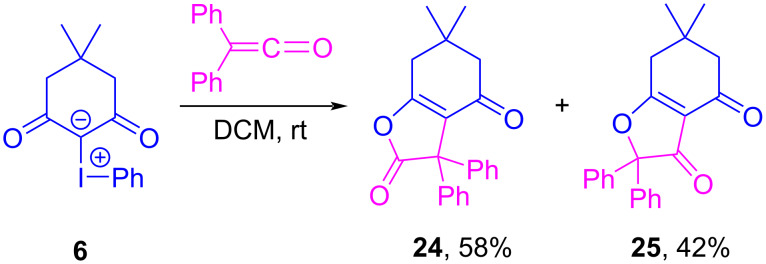
Reaction of ylide **6** with diphenylketene to form lactone **24** and **25**.

**Figure 7:**
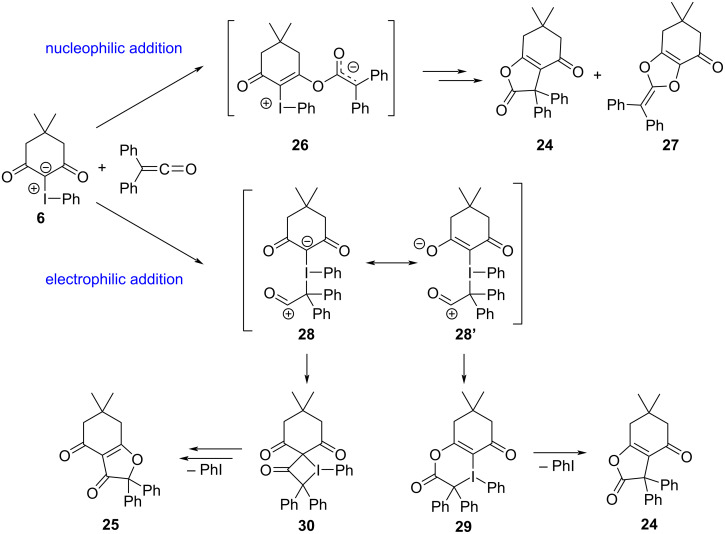
Nucleophilic (top) and electrophilic (bottom) addition pathways proposed by Koser and Hadjiarapoglou for the cyclization of **6** with diphenylketene.

Interestingly, Hadjiarapoglou also revisited their investigation of iodonium ylide cycloadditions and their associated mechanisms [120], using diphenylketene as a new reaction partner with dimedone iodonium ylide **6** (analogous to earlier work reported by Koser in 1975 [9]). Investigations by both Koser and Hadjiarapoglou were performed using identical substrates and solvents (albeit at different concentrations), and yet different product compositions were obtained. Hadjiarapoglou reported that ylide **6** reacted with diphenylketene to give **24** in 58% yield, as well as **25** in 42% yield (Scheme 4). In the earlier study, Koser recovered **24** and **27**, and they proposed an ionic mechanism wherein the ylide acted as a nucleophile, giving **26** as the initial intermediate (Figure 7, top). To account for the differing products observed, Hadjiarapoglou instead suggested that an electrophilic addition pathway was operative, producing halogen-bonded adducts **28**/**28’** which could cyclize on the acylium at either carbon or oxygen, to eventually produce **24** and **25** after reductive elimination of iodobenzene (Figure 7, bottom).

Based on these initial results and their corresponding mechanistic proposals, iodine was presumed to play a central role in these metal-free processes. Various cycloaddition reactions of iodonium ylides that were typically associated with metallocarbene-mediated processes were also operative in the absence of catalysts. The mild reaction conditions precluded researchers from proposing analogous free-carbene mediated reactions, and they instead proposed iodine to have engaged with Lewis basic ligands and coordinated their coupling within its ligand sphere. An ylide’s positively charged iodine was consistently proposed to accept ligands, via either concerted or ionic steps, ultimately leading to an iodocycle that underwent reductive elimination of an iodoarene.

#### 2.3 Halogen bonding-initiated reactions: recent discoveries

In recent years, additional processes have emerged that involve halogen bond-based adducts between iodonium ylides and Lewis bases, and with these have also emerged more complex theories as to why such adducts evolve differently. As previously stated, the strength of a halogen bond interaction is impacted by various factors (e.g., electrostatics, charge transfer abilities, dispersion), where the extent of their influence depends on both the halogen bond donor and acceptor [61]. Beyond simply considering the strength of these bonds, other characteristics of this interaction should directly impact the outcomes of the reactions that they undergo. For instance, Mulliken’s initial description of an intermolecular halogen bond was of a charge transfer complex (also known as an electron donor–acceptor complex) [121], and this bonding description has recently been used to support proposals for single electron transfer (SET) reaction pathways between iodonium ylides and various halogen bond acceptors. Alternatively, halogen-bonded complexes of iodonium ylides could lead to changes in an ylide’s UV–vis absorption profile, or to its overall basicity or nucleophilicity, both of which could lead new and unexpected reactivity.

**Scheme 5:**
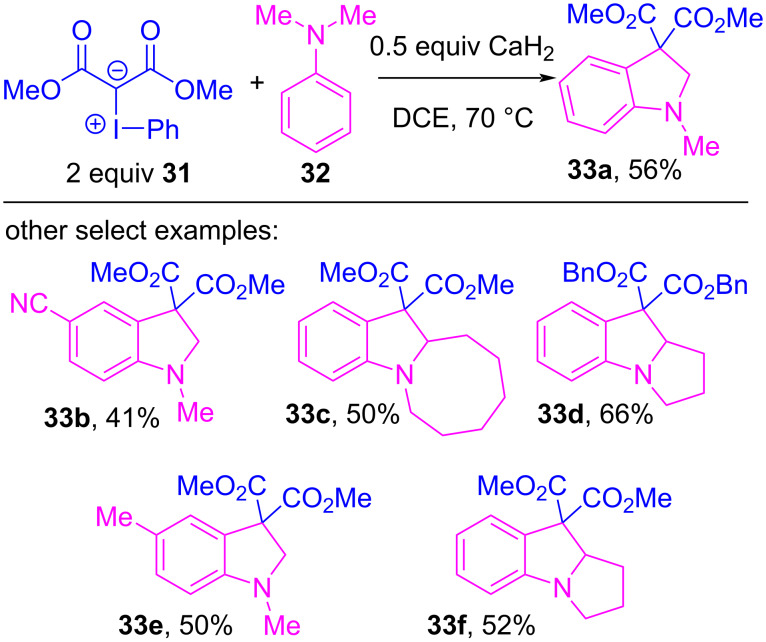
Indoline synthesis from acyclic iodonium ylide **31** and tertiary amines.

**Scheme 6:**
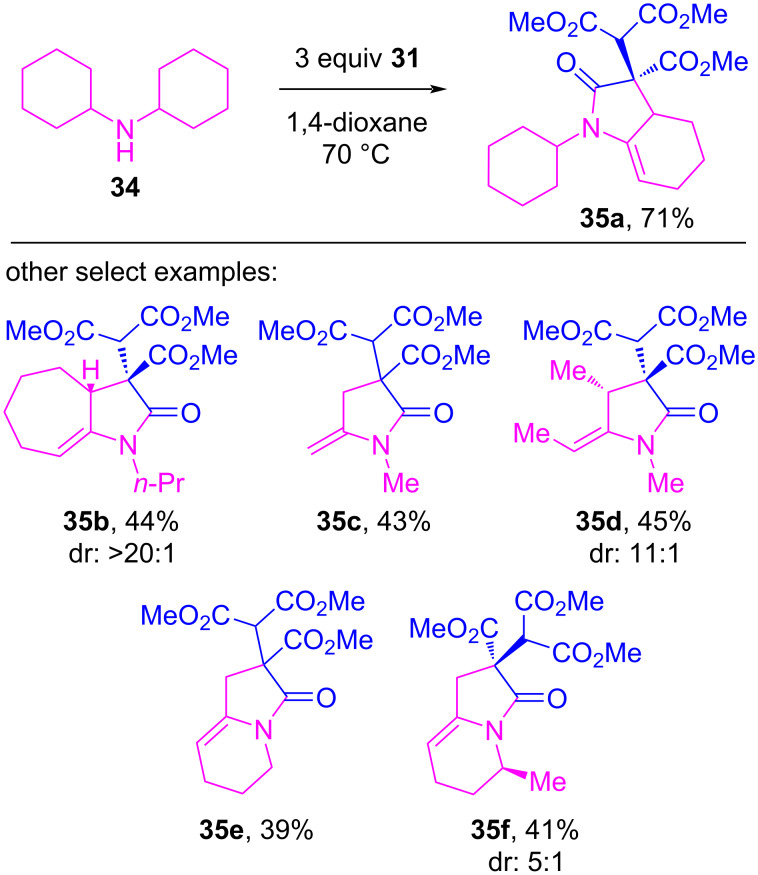
*N*-Heterocycle synthesis from acyclic iodonium ylide **31** and secondary amines.

The first example that specifically invoked electron donor–acceptor complexes of iodonium ylides was reported in 2018 by Wang and co-workers [122]. They disclosed a reaction between acyclic iodonium ylides (e.g., **31**/ **I-10**) and tertiary amines **32**, which produced indoline rings **33** by forging two new C–C bonds between the ylidic carbon and unactivated positions on the amine (Scheme 5). In 2020, they disclosed a more complex variant of this reaction that reacted **31** with secondary amines **34**, which produced densely functionalized *N*-heterocycles **35** that incorporated two of the ylide’s β-dicarbonyl motifs (Scheme 6) [123].

**Figure 8:**
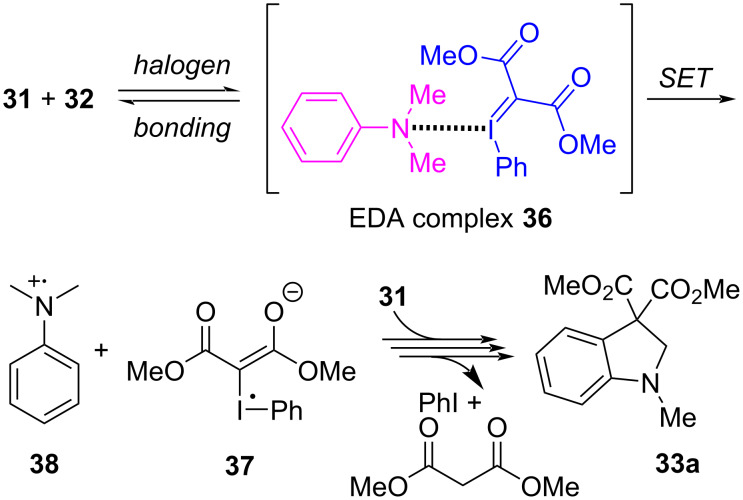
Proposed mechanism for the formation of **33a** from iodonium ylides and amines, involving an initial halogen bonded EDA complex **36**.

Though these reactions were conducted at 70 °C, free carbenes were not involved. Both TEMPO and 1,4-dinitrobenzene inhibited the formation of **33** and **35**, implying that single electron-transfer processes were occurring. Also, a competition experiment between **32** and [D_6_]-**32** gave a competitive intermolecular kinetic isotope effect of 9.5 that suggested an N-Me proton abstraction was the rate determining step. Given this, the authors proposed that electron donor–acceptor (EDA) complex **36** was initially formed between **32** and a sacrificial equivalent of **31**, and that **36** underwent a SET to give radical anion **37** and radical cation **38** (Figure 8). While one equivalent of the ylide orchestrated a series of proton transfer (PT) and SET events leading to malonate, the second equivalent of **31** coupled with the activated amine and cyclized to produce **33**. The formation of **35** in their latter study was mechanistically analogous to this proposal; however, a third equivalent of ylide **31** was necessary to facilitate an additional series of PT and SET events.

**Scheme 7:**
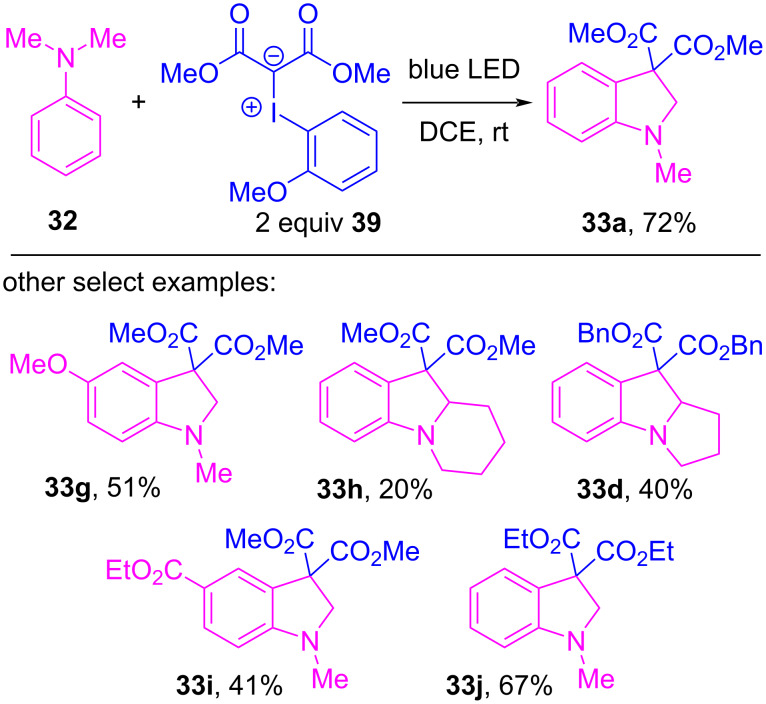
Indoline synthesis from acyclic iodonium ylides **39** and tertiary amines under blue light photocatalysis.

Xuan, Li and co-workers further investigated the coupling between iodonium ylides and tertiary amines, and showed that this thermal reaction could instead be initiated by blue light photocatalysis (Scheme 7) [124]. Therein, they investigated the iodoarene motif of the ylide, and while ylide **31** gave **33a** in 32% yield, significantly lower than the previously obtained 56%, when the *ortho*-anisyl ylide **39** was used it gave **33a** in a much improved 72% yield. The yields of other reactions that employed various amine and ylide derivatives were consistent with those previously obtained (Scheme 5). Control experiments showed that the reaction failed in the dark at room temperature, and they concluded that blue light activation of the initially-formed EDA complex (analogous to **36**) promoted the onset of SET events. As this latter protocol also required two equivalents of ylide **39**, the authors proposed a near identical mechanism to that of Wang (Figure 8). Strong evidence for formation of an initial EDA complex was found when measuring the UV–vis absorption spectra of **39**, alone and in the presence of **32**. The latter showed a significant absorption red shift, which implied a structural change in **39** had occurred, which was attributed to halogen bond complex formation.

**Scheme 8:**
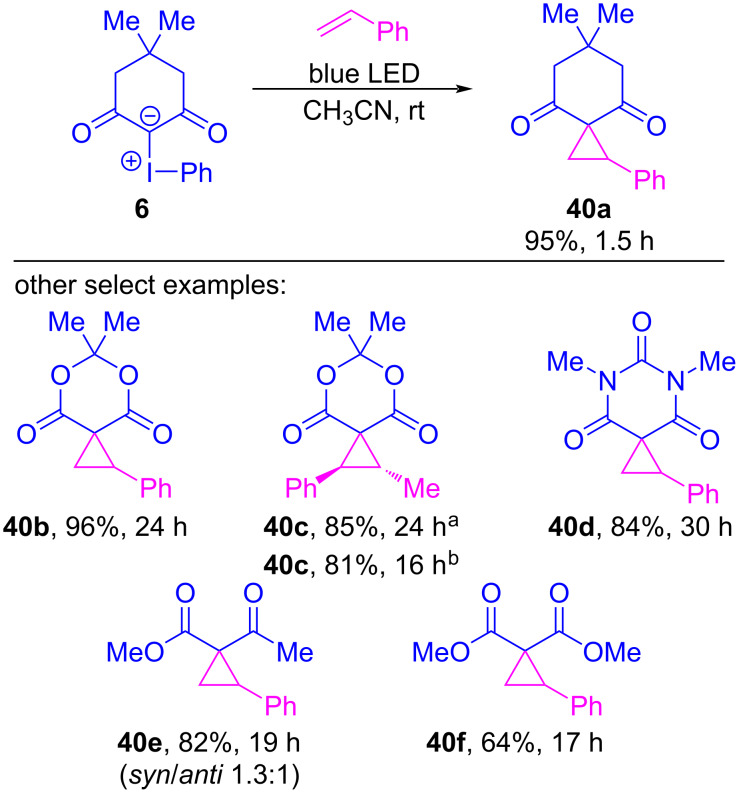
Metal-free cycloproponation of iodonium ylides under blue LED irradiation. ^a^Using *trans*-β-methylstyrene. ^b^Using *cis*-β-methylstyrene.

**Figure 9:**
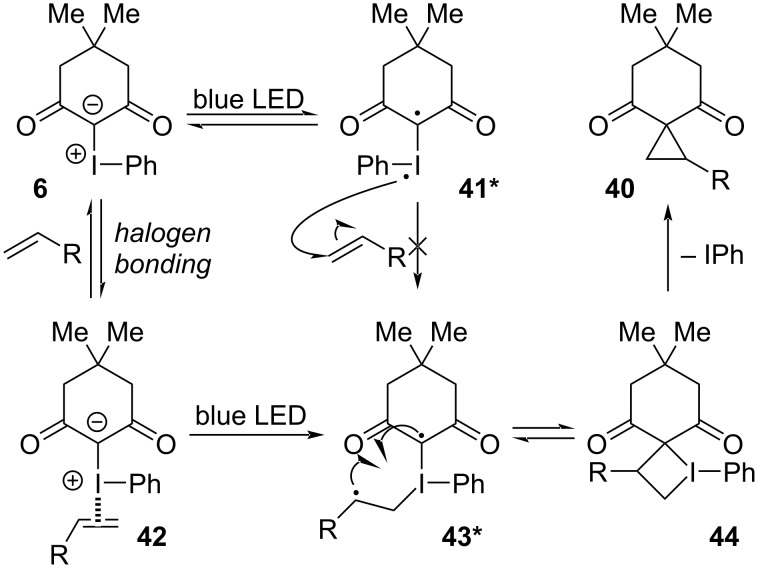
Proposed mechanism of the cyclopropanation between iodonium ylides and alkenes under blue LED irradiation.

In 2019, the Murphy group reported the first transformation induced by blue LED irradiation of cyclic and acyclic iodonium ylides (e.g., **6**) and alkenes, which generated cyclopropanes **40** in yields up to 96% (Scheme 8) [125]. UV–vis absorption spectra of various ylides were generated both computationally and experimentally, and these revealed that ylide excitation should occur in the blue light region, inducing a HOMO–LUMO excitation within the ylide. The LUMO was a molecular orbital situated primarily on iodine, and its natural population analysis revealed that 0.26 *e* of electron density was transferred to iodine, resulting in a 1,2-diradical (41*, Figure 9). In their mechanistic proposal, they presumed that this excited species would not have been sufficiently long-lived to encounter the styrene (41*→43*). Instead, they invoked an initial halogen-bonded complex **42**, whose irradiation with blue light was proposed to produce acyclic excited state 43*, where possible bond rotation could account for the convergence of alkene isomers into a single product (**40c**). Radical coupling would then lead to the typical iodocyclobutane intermediate **44**, from which reductive elimination of iodobenzene would generate **40**.

**Scheme 9:**
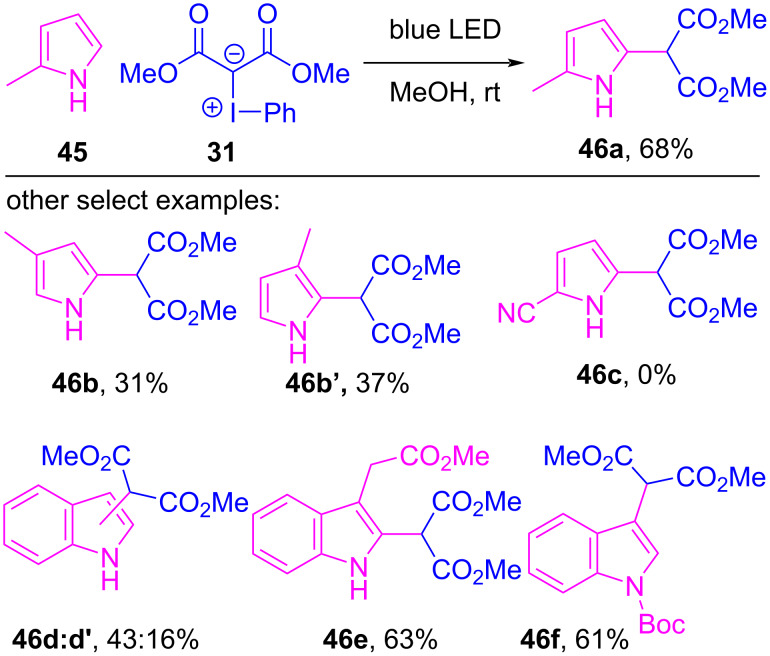
Formal C–H alkylation of iodonium ylides by nucleophilic heterocycles under blue LED irradiation.

**Figure 10:**
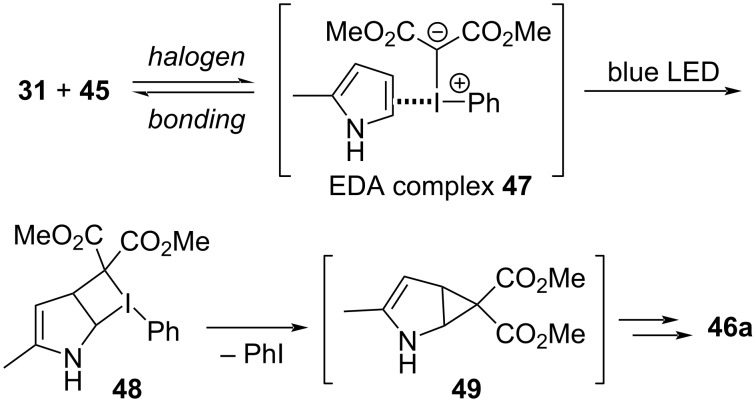
Proposed mechanism of the formal C–H insertion of pyrrole under blue LED irradiation.

In 2021, Sen and Gremaud disclosed a blue LED-mediated formal C–H insertion reaction between iodonium ylides (e.g., **31**) and pyrroles (e.g., **45**), indoles and furans, producing malonate-substituted heterocycles **46** (Scheme 9) [126]. The authors discounted a free carbene-based C–H insertion because conducting the reaction in the presence of the radical trap phenyl *N-tert*-butyl nitrone (PBN) and the radical scavenger TEMPO resulted in decreased yields and isolation of their iodonium ylide adducts. Additional kinetic isotope effect studies revealed that the C2–H bond of pyrrole did not participate in the rate-determining step. This led the authors to propose a multi-step reaction mechanism, where irradiation of an initial halogen-bonded EDA complex **47** led directly to iodocyclobutane **48** (Figure 10). Reductive elimination of iodobenzene then generated a strained, cyclopropane-fused bicycle **49** that rearranged into the isolated C–H insertion product **46a**.

**Scheme 10:**
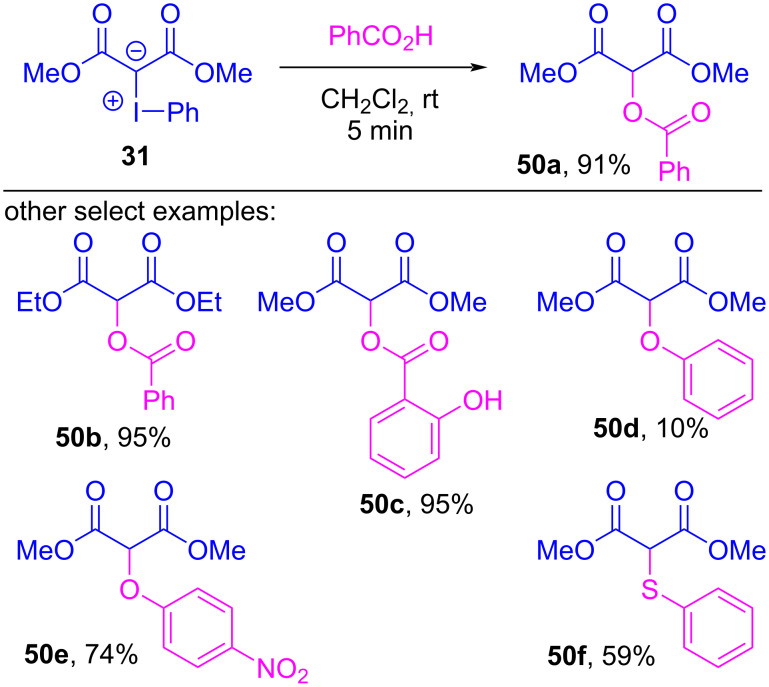
X–H insertions between iodonium ylides and carboxylic acids, phenols and thiophenols.

**Figure 11:**
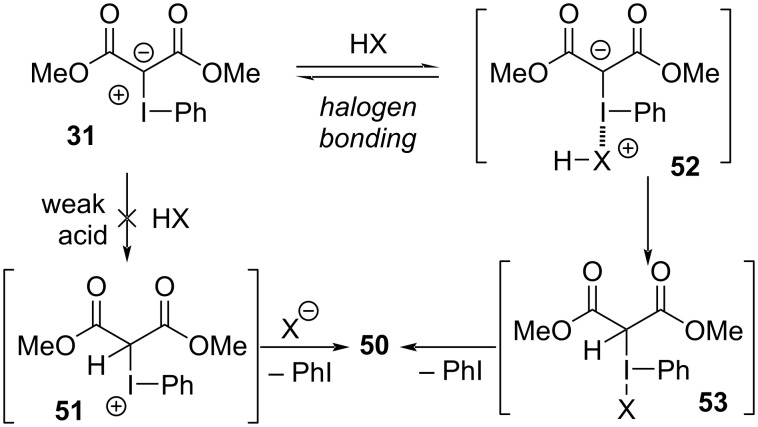
Mechanistic proposal for the X–H insertion reactions of iodonium ylides.

In addition to Neiland’s disclosure of the reactions between iodonium ylides and hydrochloric acid (Scheme 1) [1,102], others have also reported that both strong acids (e.g., TsOH, TFA, and trichloroacetic acid) and even weak acids (e.g., HF, Et_3_N·3HF, and AcOH) react with iodonium ylides to give formal X–H insertions [127–130]. In 2022, Murphy et al. disclosed a computational and experimental investigation into the reaction scope and the mechanism by which such X–H insertion reactions could occur (Scheme 10) [100]. Systematic evaluation of acidic compounds showed that carboxylic acids (p*K*_a_ ≈ 5; 85–99% yield) and thiophenols (p*K*_a_ ≈ 7; **50f**, 59%) were especially viable, whereas electron-rich phenols (p*K*_a_ ≈ 10; **50d**, <15% yield) were not. Though electron-poor phenols (p*K*_a_ ≈ 8; **50e**, 64–74% yield) were suitable, they were unreactive in the presence of a carboxylic acid (**50c**). If these insertions proceed via initial protonation of the iodonium ylide to produce **51** (Figure 11), its high acidity (p*K*_a_ ≈ 0) [131–132] would suggest that only strong acids should undergo this reaction; however, the reaction was also viable with weakly acidic compounds (p*K*_a_ < 8). There was no evidence of free carbenes being involved, and competition experiments showed that both electron-rich and electron-poor benzoic acids reacted faster than benzoic acid, which suggested that both proton acidity and the Lewis basicity of the X–H molecule were important. This led the authors to propose an initial halogen-bonded complex **52** (consistent with the rate acceleration seen for more Lewis basic reactants), which would weaken the X–H bond and simultaneously strengthen the basicity of the ylidic carbanion. Transfer of the proton would give **53**, from which expulsion of iodoarene would generate the formal X–H insertion products **50**.

In these recent reports, researchers commonly invoked EDA complexes between iodonium ylides and their Lewis basic reaction partners. This served to pre-complex reactants such that they can undergo intramolecular reactions within the iodine’s ligand sphere. These EDA complexes have been proposed to then undergo single electron transfers from the Lewis base to the ylide, under both thermal or blue LED irradiation conditions, leading to C–H insertion products. Irradiating EDA complexes between alkenes and iodonium ylides with blue light was believed to induce intramolecular iodocyclobutane formation, followed by cyclopropane formation. These reactions were believed to either involve 1,2-diradicals on the ylide, which could engage the complexed alkene, or to involve direct cycloaddition between the ylide and alkene. Murphy’s report of formal X–H insertions with iodonium ylides was similarly proposed to initiate upon complex formation with a Lewis base. Adduct formation was believed to both increase the acidity of halogen bond acceptor’s attached protons, as well as increase the basicity of the ylide’s β-dicarbonyl motif, thereby enabling unexpected reactivity. The reactions available to iodonium ylides have extended well beyond the traditional roles of metallocarbene precursors, where halogen bonding is a key element of modern mechanistic proposals.

#### 2.4 Radiofluorination of iodonium ylides

**Scheme 11:**
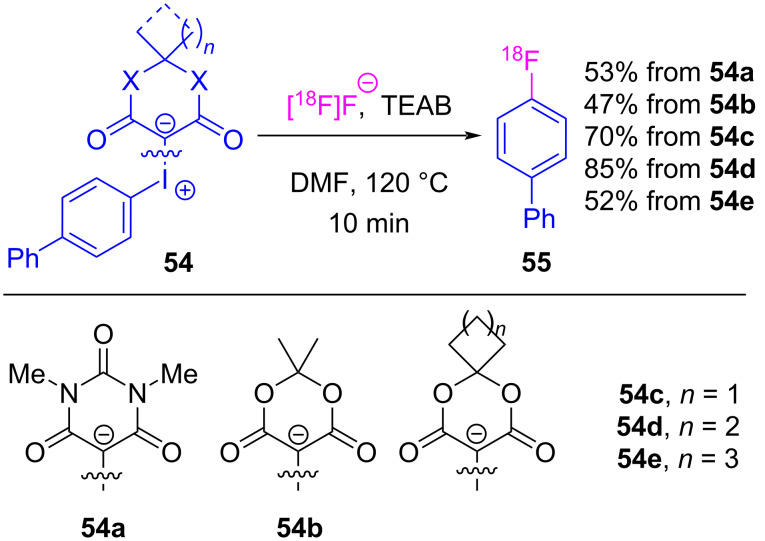
Radiofluorination of biphenyl using iodonium ylides **54a**–**e** derived from various β-dicarbonyl auxiliaries.

**Scheme 12:**
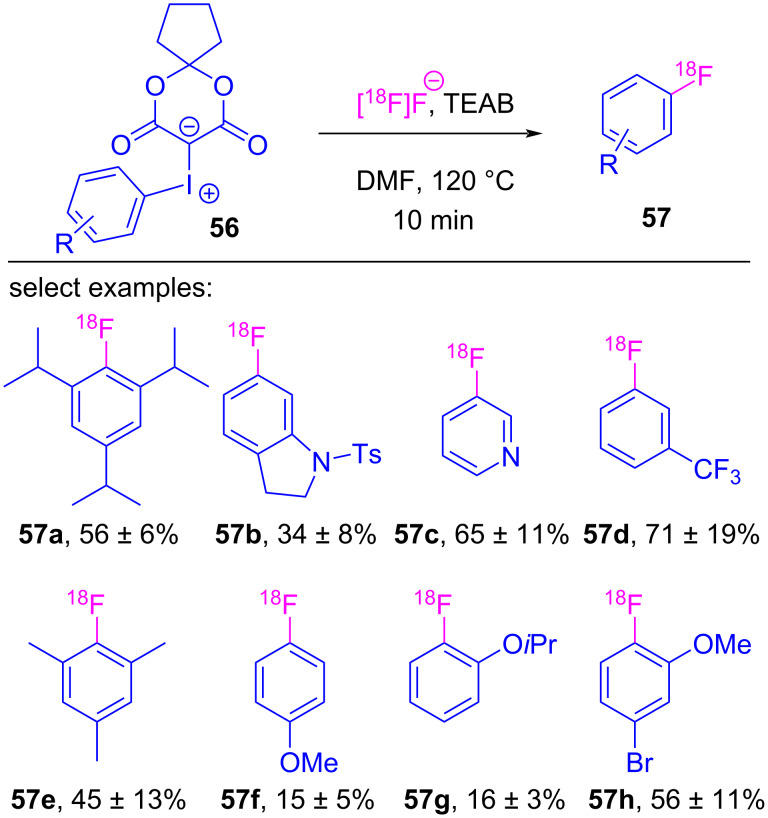
Radiofluorination of arenes using spirocycle-derived iodonium ylides **56**.

**Scheme 13:**
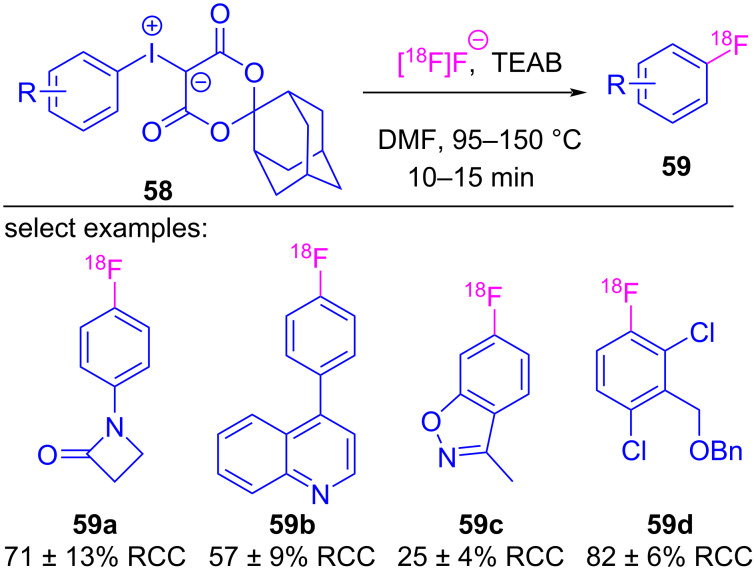
Radiofluorination of arenes using SPIAd-derived iodonium ylides **58**.

In the preceding sections, the halogen-bonded complexes between iodonium ylides and Lewis basic reactants all decomposed to furnish functionalized β-dicarbonyl motifs. This is despite there being two available σ-holes for complex formation, and two pathways by which reductive elimination (e.g., with the β-dicarbonyl or arene) could occur (see Scheme 1). A prominent example of the coupling occurring between the Lewis base and the arene is in reactions with fluoride, which has been translated to enable the radiofluorination of non-activated arenes. While this fluorination reaction was first reported in 2010 [133], the subsequent collection of works by Vasdev and Liang led to an improved methodology appropriate for the time and temperature requirements of radiofluorination reactions, and led to a mechanistic proposal dependent on σ-hole selective halogen bonding [134]. Vasdev and Liang modified the iodonium ylide’s β-dicarbonyl skeleton (Scheme 11), and while derivatives of barbituric acid (**54a**) and Meldrum’s acid (**54b**) were moderately effective, replacing the *gem*-dimethyl with spirocyclic motifs **54c**–**e** provided [^18^F]fluorobiphenyl **55** in 47–85% yield [134]. Decomposition studies showed that this spirocyclic alkyl motif increased the stability of the ylide, which was essential given the elevated temperatures used in radiofluorination. They then prepared and tested a series of spirocyclopentane-derived ylides **56**, and were able to radiofluorinate a series of electron-rich and electron-poor arenes **57**, including hindered *ortho*-substituted derivatives (Scheme 12). Further derivatization of the spirocyclic motif led to SPIAd-derived iodonium ylides **58**, whose radiofluorinations showed improved radiochemical conversions with a variety of arene derivatives (Scheme 13) [16].

**Figure 12:**
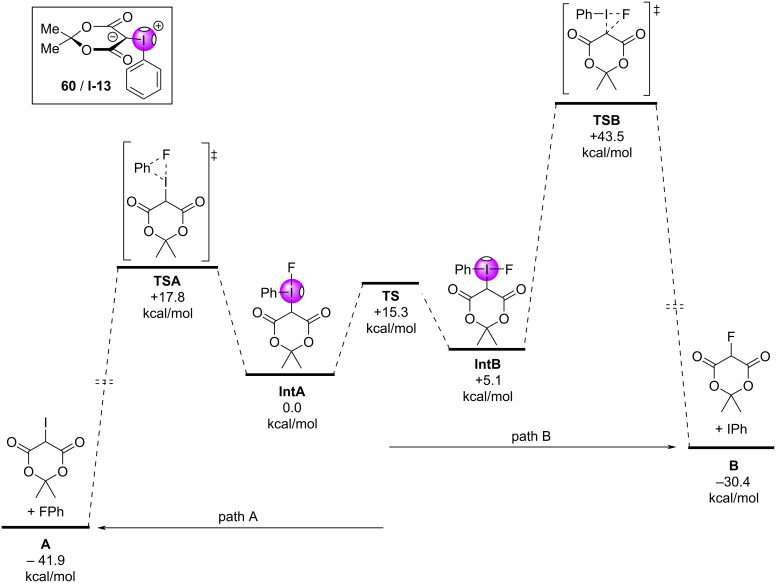
Calculated reaction coordinate for the radiofluorination of iodonium ylide **60**.

Computational investigations were conducted to better understand these reactions, and it was determined that the changing alkyl motif (e.g., dimethyl, cyclopentyl, adamantyl) had minimal impact on the activation energy of the fluorination reactions. The reaction coordinate was calculated for Meldrum’s acid-derived iodonium ylide (**60**/**I-13**) (Figure 12, inset), which showed a 5.1 kcal/mol energy difference between rapidly equilibrating halogen-bonded fluoride adducts. The lower energy adduct **IntA** had fluoride residing in the stronger σ-hole opposite the β-dicarbonyl (*syn* to the arene), whereas the higher energy adduct had fluoride residing in the weaker σ-hole (*syn* to the β-dicarbonyl) [16]. These calculations showed that a reductive elimination event was operative from both **IntA**/**IntB**, with a >25 kcal/mol energy difference between transitions states of the observed fluorination (**TSA**, path A) versus that of the alternate Csp^3^–F reductive elimination pathway (**TSB**, path B). These computations are consistent with all known experimental observation, as over 100 iodonium ylides have been tested in these fluorination reactions and none have reacted via this alternative decomposition pathway.

**Scheme 14:**
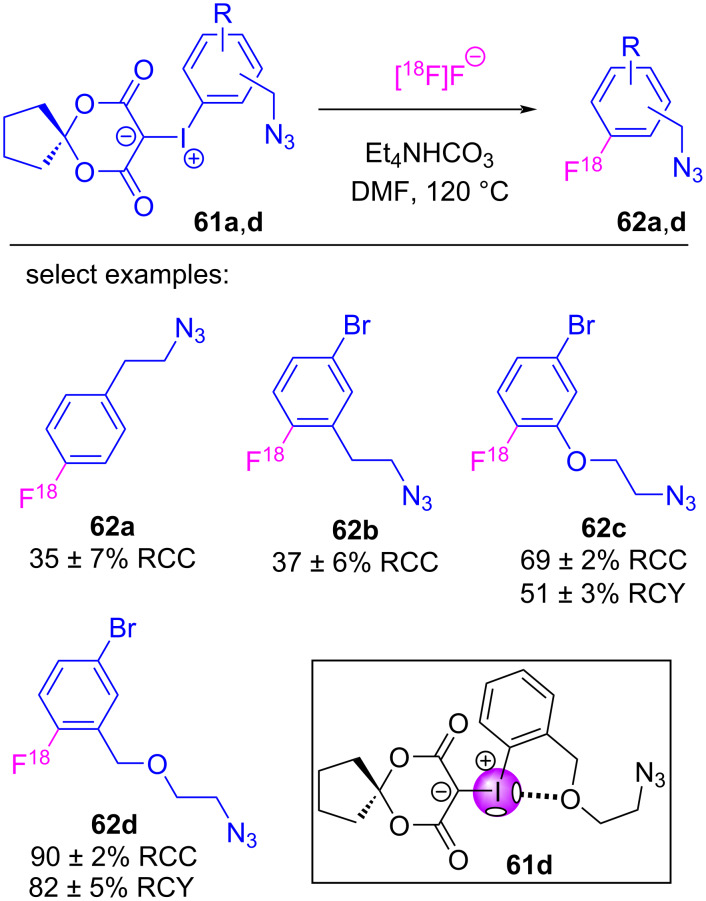
Radiofluorination of iodonium ylides possessing various *ortho-* and *para-*substituents on the iodoarene.

Vasdev and Liang also assessed the effect of *ortho*-substituents on iodonium ylides undergoing radiofluorination, similar to that encountered with diaryliodonium salts [135–138]. They prepared a series of ylides (**61a**–**d**) with substituents situated *ortho-* or *para*- to iodine, and found the radiochemical conversions (RCCs) of the *ortho-* or *para*-alkyl derivatives to be consistent at 37% (**62b**) and 35% (**62a**), respectively (Scheme 14) [139]. However, when an *ortho*-alkoxy substituent (**61c**) was used, a significantly improved 69% RCC (51% radiochemical yield) was achieved for **62c**, despite it being derived from an electron-rich, deactivated arene. Improved results were further realized for **61d**, in which the oxygen was one atom removed from the arene, which gave **62d** in a 90% RCC and an 82% RCY. These results offered important insight into the hierarchy of fluorination selectivity and pointed directly to secondary bonding occurring between the Lewis basic ether and a σ-hole on the electropositive iodine (Scheme 14, inset). These results suggest that with iodonium ylides, *ortho*-substituents impose an electronic effect (compare **62a** vs **62b** and **62b** vs **62c**), contrary to the steric effect observed for radiofluorination of diaryliodonium salts [136,140]. Incorporation of a Lewis basic ether that could participate in secondary bonding with iodine was critical, and further improvements were realized when this Lewis base was not deactivating the ring (**62c** vs **62d**).

**Figure 13:**
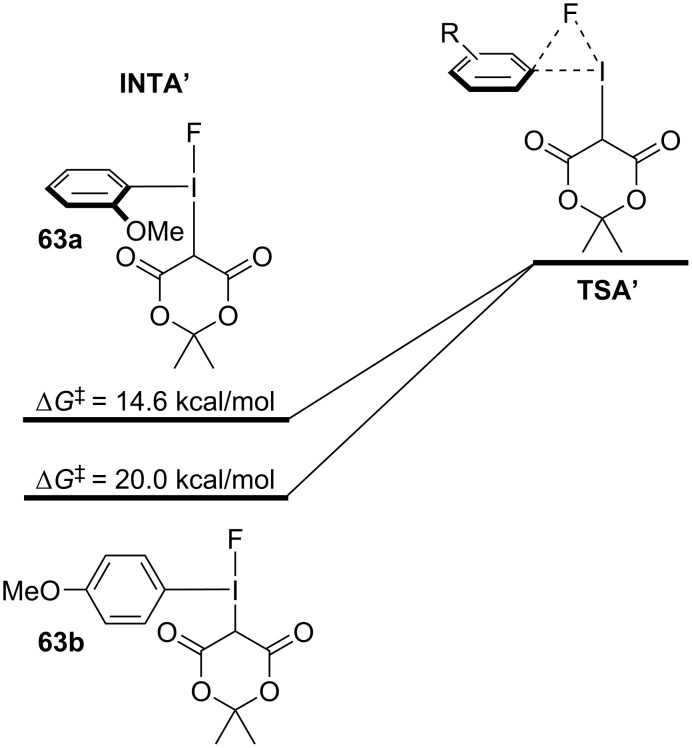
Difference in Gibbs activation energy for *ortho*- or *para*-anisyl derived iodonium ylides **63a** and **63b**.

**Figure 14:**
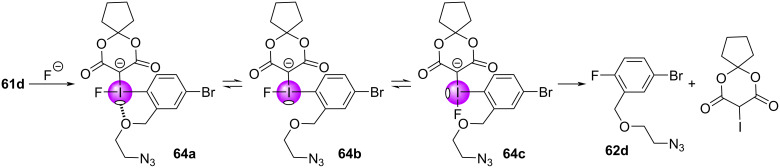
Proposed equilibration of intermediates to transit between **64a** (the initial adduct formed between **61d** and fluoride) and **64c**, to properly situate fluorine for reductive elimination.

From these examples, the evidence suggests that σ-holes are playing an important role in the outcomes of (radio)fluorination reactions. Computational evidence suggests an energetic preference for fluorine adducts with the stronger σ-hole, reminiscent of hard/soft acid and base principles, where better electrostatic stabilization is achieved when the hard fluoride interacts with the stronger σ-hole. Structural modification of the β-dicarbonyl auxiliary has led to improved outcomes, due to increased ylide stability rather than decreasing activation energies of the fluorination reaction. There are, however, other conflicting pieces of evidence that indicate these reactions are much more complex. For example, in earlier computational studies of anisyl-derived iodonium ylides **63a/b**, Vasdev and Liang found a steric explanation for the *ortho-*substituent effect (Figure 13), contrary to that observed for **62a/b**. They showed how the *ortho*-methoxy group in **63a** induced an energetically-disfavoured twist in the fluorine adduct’s conformation (**INTA’**), raising its energy over that of **63b** and decreasing the energy barrier between it and the reductive elimination transition state **TSA’**. Additionally, the role of the halogen bond acceptor in **61d** is also poorly understood. Intramolecular halogen bonds might be expected to block the proximal (strong) σ-hole and prevent fluoride addition at that site (**64a**, Figure 14); however, computational results suggested that adduct formation at the strong σ-hole was key to the chemoselective reductive elimination of the fluoroarene. From this, we might infer that adduct equilibration was occurring (e.g., **64b**,**c**) to properly situate the fluorine prior to the reductive elimination event (refer to Figure 14).

#### 2.5 Intramolecular halogen bonding in iodonium ylides

Halogen bonding was encountered as numerous intra- and intermolecular processes throughout the preceding sections, and was often formed between the ylide and the intended nucleophile (e.g., alkene, amine or halide) of the reaction. However, intramolecular halogen bonding was also observed with other non-reacting Lewis bases, where it altered an ylide’s stability and even impacted the chemoselectivity of its subsequent reactions. Examples of this include the *ortho*-substituted iodoarene-derived ylides used in blue LED photoreactions between ylides and amines (Scheme 7) and radiofluorinations (Scheme 14), where *ortho*-ether moieties positively influenced the reaction’s outcome. Additional examples of intramolecular secondary interactions also exist in the literature, in which *ortho*-substituents on iodoarene-derived ylides have been key to tuning reactivity and improving reaction outcomes.

**Scheme 15:**
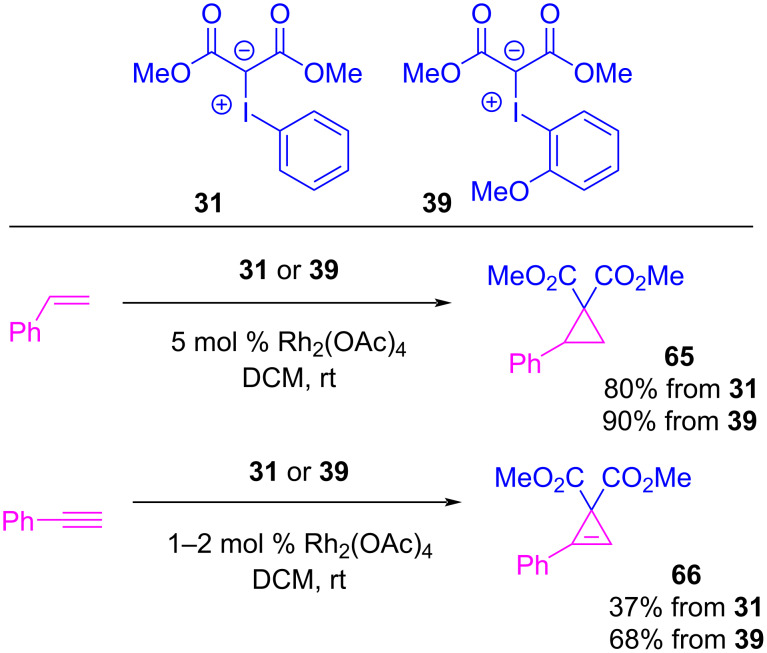
Comparison of **31** and *ortho*-methoxy iodonium ylide **39** in rhodium-catalyzed cyclopropanation and cyclopropenation reactions.

**Figure 15:**
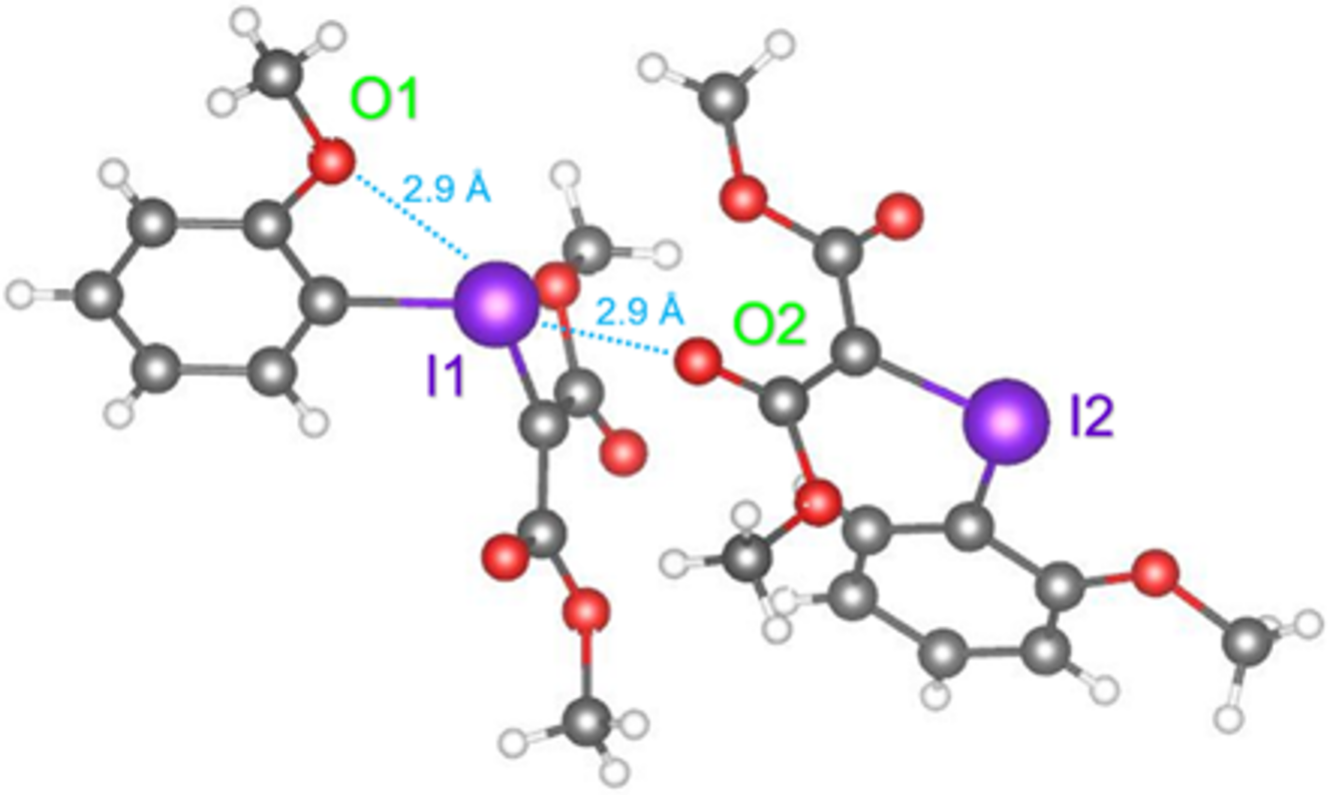
X-ray crystal structure of dimeric **39** [6], (CCDC# 893474) [143–144].

Zhdankin et al. first reported on this concept, testing *ortho*-alkoxy-substituted iodoarene-derived iodonium ylides for improved solubility and stability [5–6,141]. *ortho*-Iodoanisole-derived ylide **39** was subject to a rhodium-catalyzed cyclopropanation reaction with styrene, and the results were compared with Charette’s 2009 work on **31** (Scheme 15) [142]. While both ylides were effective as metallocarbene precursors, the *o*-anisyl derivative **39** gave an improved 90% yield of **65**, compared to the 80% yield achieved with ylide **31** under identical reaction conditions. Zhdankin also assessed the cyclopropenation of phenylacetylene, in which **39** gave **66** in 68% yield, which was again a significant improvement over the 37% achieved by Müller in 1995 with ylide **31** [3]. The crystal structure of **39** was also reported and it showed clear secondary halogen bonding that engaged both of iodine’s σ-holes, as both intramolecular (2.9 Å I1–O1) and intermolecular (2.9 Å (I1–O2) interactions (Figure 15). The authors’ explanation for the improved reactivity of *o*-anisyl derivative **39** was based on its improved solubility in dichloromethane. The contact distances were both within the Van der Waals radii criteria for confirming non-covalent interactions [33], which likely contributed to the ylide’s increased stability. As the Lewis basic *ortho*-methoxy was engaged with the ylide’s stronger σ-hole, it likely resulted in weaker intermolecular interactions that diminished its aggregation ability and increased its solubility. However, the full impact of this *ortho*-methoxy group was unclear, as it could also have improved the nucleophilicity of the carbanion (see **52**, Figure 11), facilitating the initial metallocarbene forming event.

**Scheme 16:**
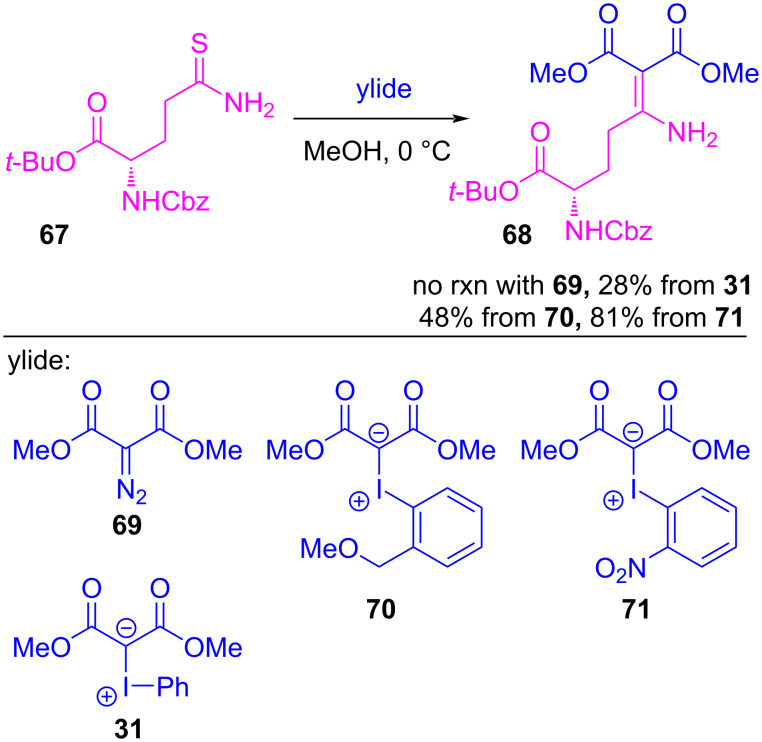
Enaminone synthesis using diazonium and iodonium ylides.

In 2019, Takemoto et al. investigated *ortho*-groups as a means to enhance the stability and solubility of ylide precursors, and they envisioned how blocking one of iodine’s σ-holes might enhance the reaction’s chemoselectivity. Specifically, they investigated the coupling reactions between diazonium and iodonium ylides and the soft Lewis base thioamide **67** [145], intending to achieve C–S bond formation with the β-dicarbonyl, producing **68** (Scheme 16). They compared a series of ylide precursors and found that when diazo compound **69** was reacted with **67** (with or without a transition-metal catalyst), no reaction occurred. Conversely, iodonium ylides **31**, **70** and **71** all reacted with **67** to produce **68** in 28–81% yield. A significant improvement was realized when an *ortho*-ether substituent was present (**31** vs **70**), and the highest yield was found with *ortho*-nitro ylide derivative **71**.

**Figure 16:**
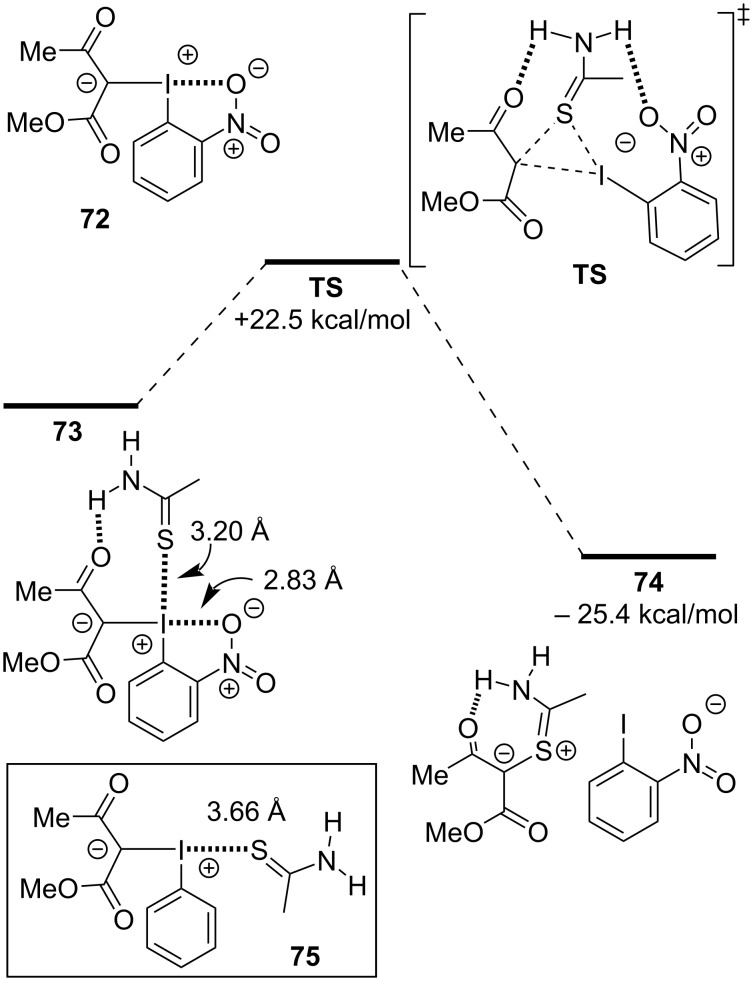
Transition state calculations for enaminone synthesis from iodonium ylides and thioamides.

Computational studies, using abridged ylide **72** and thioacetamide as model substrates, were also performed to better understand the mechanism, specifically exploring the role of the nitro group and the reductive elimination of the iodoarene (Figure 16). They found **72** to possess secondary intramolecular halogen bonding between the nitro group and iodine’s proximal (stronger) σ-hole. The reaction initiated by selectively forming adduct **73** between the thioamide and the ylide, which possessed both halogen- and hydrogen intermolecular bonds. This adduct decomposed by reductive elimination of the iodoarene via a three-membered transition state (**TS**), coupling the *syn* thioamide and β-dicarbonyl ligands to give thiocarbenium ion **74**. The authors determined similar activation energies (≈20 kcal/mol) when comparing ylides **31** and **70** in this reductive elimination step, which suggested that the *ortho*-nitro group of **72** (and **71**, by extension) played a role other than lowering the activation energy of this step. Given that other optimized halogen- and hydrogen-bonded conformations were found between thioamide and these ylides (e.g., **75**), they surmised that the nitro group was best suited to blocking the proximal σ-hole, thereby preventing non-productive reaction pathways (e.g., thioamide arylation) from occurring.

**Scheme 17:**
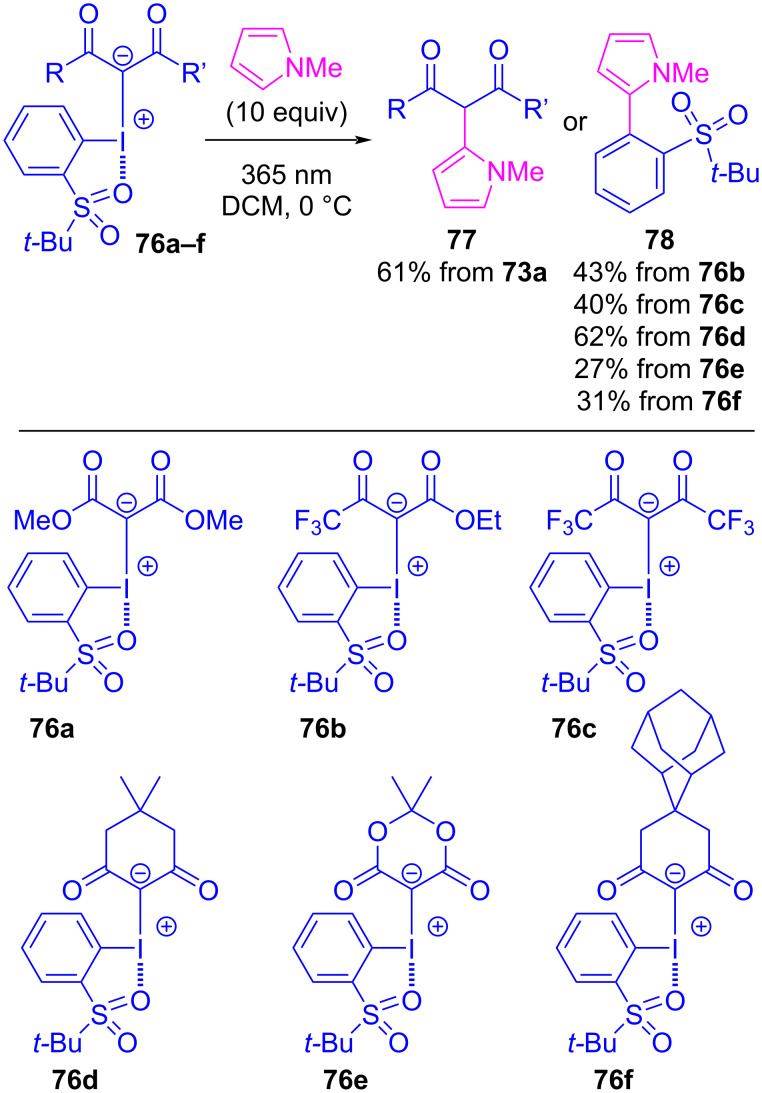
The reaction between ylides **73a**–**f** and *N-*methylpyrrole under 365 nm UV irradiation.

**Figure 17:**
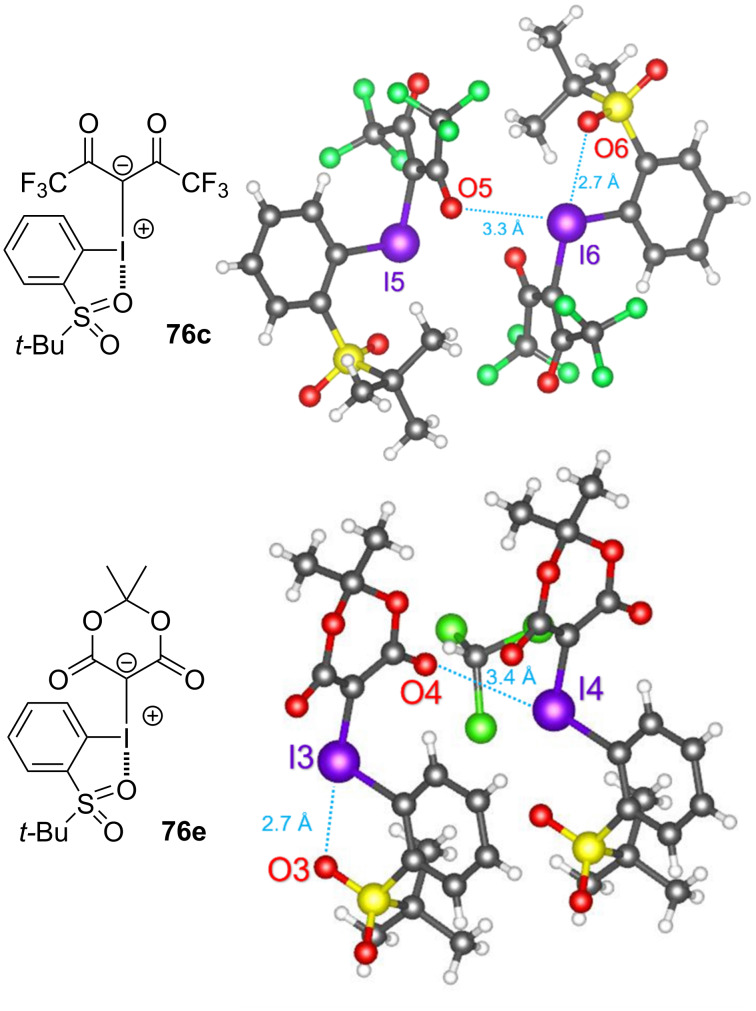
Crystal structures of **76c** (top) and **76e** (bottom) [101], (CCDC# 2104180 & 2104181) [143–144].

Takemoto and Kobayashi have theorized that an equilibrium should exist between Lewis-basic nucleophiles and iodonium ylides, where the nucleophile could halogen bond to either σ-hole, and that this could lead to distinct reductive elimination pathways (see Scheme 1) [101]. To probe this theory, they prepared a series β-dicarbonyl-derived iodonium ylides (**76a**–**f**) possessing a σ-hole-blocking *ortho*-*t*-BuSO_2_ group on the iodoarene (Scheme 17). A natural-bond-orbital (NBO) analysis conducted on hexafluoroacetylacetone-derived ylide **76c** identified three distinct lone pair interactions between the sulfonyl oxygens and the σ* orbital of the I–C bond, at energies of 0.79, 3.15, and 4.73 kcal/mol. Also found were intermolecular interactions between the lone pairs of either the carbonyl or sulfonyl with the other I–C bond σ* orbital, at energies of 0.70 and 0.29 kcal/mol, respectively. These interactions were presumed to have contributed to the increased stability of such ylides. X-ray crystal structures of ylides **76c** and **76e** confirmed these interactions as intramolecular halogen bonds at lengths of 2.7 Å (Figure 17), and showed intermolecular halogen bonds with iodine’s remaining σ-hole, at bond lengths of 3.3 Å or 3.4 Å. Though the σ-hole *syn* to the arene was blocked, when these six ylides were subject to photoirradiation with *N*-methylpyrrole, products corresponding to both β-dicarbonyl- (**77**, expected) and arene- (**78**, unexpected) functionalization were observed. When malonate-derived ylide **76a** was reacted with *N*-methylpyrrole, it produced **77a** in 61% yield. This presumably occurred via a reaction sequence analogous to that proposed by Sen and Gremaud for reactions between ylides and 2-methylpyrrole, involving irradiation of an initially-formed EDA complex (see Figure 10). However, the remaining ylides **76b**–**f** all reacted to produce arene-coupled **78b**–**f** as the sole product in 27–62% yield. The formation of these two product classes supports their hypothesis that two halogen bond-initiated reactions are possible with such ylides. As the σ-hole *syn* to the arene was blocked, one might have expected these ylides to be predisposed to forming **77** exclusively, but this was not observed. Similarly, one might have expected these ylides to display consistent chemoselectivity, but this was also not observed. The reasons for this “anomalous” reactivity remain unclear, as it is unprecedented that changing the β-dicarbonyl motif of an ylide would flip its ligand coupling selectivity. This could be attributed to a range of factors including disfavoured sterics in the reductive elimination or higher energy UV photoirradiation enabling unknown reactivity pathways, and it points to an unknown effect of changing an ylide’s β-dicarbonyl motif. Nonetheless, these results represent the first examples (beyond halogenation) where the arene of an ylide has undergone coupling with a Lewis basic reactant.

Collectively, these results show the clear benefits of intramolecular halogen bonding in achieving increased solubility and stability of iodonium ylides. When Lewis basic motifs were incorporated *ortho-* to the iodine on the arene, these engaged with the ylide’s stronger σ-hole to block this site and diminish the ylide’s aggregation potential. However, intramolecular σ-hole blocking by halogen bond acceptors appears to be reversible, as these σ-holes do participate in reactions with extraneous Lewis basic reactants, presumably via equilibration between initially-formed adducts. And while the impact of these halogen bond acceptors on subsequent mechanistic events remains unknown, these represent an easily modified structural feature that may prove instrumental to discovering new reactivity patterns for iodonium ylides.

## Conclusion

Iodonium ylides are an important subset of hypervalent iodine compounds [146–148], and these have received increased attention from the synthetic community over the past decade. In addition to conventional cycloaddition or metallocarbene-based reactions of iodonium ylides, their scope has recently expanded to include X–H insertions based on single electron transfer (SET) mechanisms, cycloadditions based on blue LED photochemistry and radiofluorinations of unactivated arene motifs. In these reports, mechanistic proposals commonly treat the hypervalent iodine atom as being metal-like, orchestrating ligand couplings within its ligand sphere. Iodonium ylides were recently confirmed to possess two σ-holes of differing strengths, and theories about these ylides react now explicitly invoke halogen bonding and EDA complexes between iodine and Lewis basic reactants. Many of these theories are still in their infancy, including whether hard/soft acid and base principles may be relevant to matching σ-hole selectivity (such as in radiofluorination), and whether the *ortho-*Lewis base effect is merely one of ylide stabilization, or if this also influences σ-hole selectivity and ultimately the reaction’s chemoselectivity. Through this review, we hope to have given researchers a concise summary of halogen bonding in iodonium ylides, how they are operative in their mechanisms, and provided a compelling case for continued investigation of this versatile compound class.
